# Generative Adversarial Network Performance in Low-Dimensional Settings

**DOI:** 10.6028/jres.126.008

**Published:** 2021-04-20

**Authors:** Felix Jimenez, Amanda Koepke, Mary Gregg, Michael Frey

**Affiliations:** 1 National Institute of Standards and Technology, Gaithersburg, MD 20899, USA; 2 University of Colorado Boulder, Boulder, CO, 80309 USA

**Keywords:** earth mover distance, experiment protocol, generative adversarial network, mode tunneling, modeling error, target distribution complexity

## Abstract

A generative adversarial network (GAN) is an artifcial neural network with a distinctive training architecture, designed to create examples that faithfully reproduce a target distribution. GANs have recently had particular success in applications involving high-dimensional distributions in areas such as image processing. Little work has been reported for low dimensions, where properties of GANs may be better identifed and understood. We studied GAN performance in simulated low-dimensional settings, allowing us to transparently assess effects of target distribution complexity and training data sample size on GAN performance in a simple experiment. This experiment revealed two important forms of GAN error, tail underflling and bridge bias, where the latter is analogous to the tunneling observed in high-dimensional GANs.

## 1 Introduction

A generative network is an artificial neural network (ANN) designed to synthesize examples similar in desired ways to the examples on which the network is trained. The examples presented to the generative network during training are understood to be points in a sample *S* drawn from a target distribution *T*, and the generative network seeks to learn *T* . Because the learning process is imperfect, the target distribution *T* and the distribution *L* actually learned by the generative network will differ, and this error—the distance *d*(*T, L*) between the two distributions—determines the generative network’s performance. An example of generative modeling is the task of creating faces for cartoon characters [1]. A generative network is trained with examples of cartoon faces taken from available hand-drawn animations so that it can synthesize faces that are original but yet, for example, have the desired hand-drawn appearance and adherence to face conventions (nose above mouth, etc.). The target distribution *T* in this example is the population of all cartoon faces with the desired characteristics. Based on a sample *S* of training faces from *T*, the generative network learns to synthesize faces with distribution *L*, ideally with an error *d*(*T, L*) so small that a face randomly generated from *L* and a target face from *T* are indistinguishable in the desired ways.

Generative adversarial networks (GANs), introduced by Goodfellow *et al*. [2] and Creswell *et al*. [3], are a form of generative network with a novel training architecture in which the generative network, termed the generator, is trained alongside a classifier network, termed the discriminator. In this arrangement the generator and discriminator are pitted one against the other and trained simultaneously in iterative fashion: At each iteration the generator presents learned examples to the discriminator, and the discriminator attempts to correctly identify these learned examples from among a pool of training examples. The discriminator reports its successes and failures back to the generator, each network updates itself based on the discriminator’s successes and failures, and a new iteration begins. At each iteration the generator is trying to mislead the discriminator, and the discriminator is trying to avoid being misled. After enough iterations, the generator and discriminator approach a game-theoretic equilibrium where the discriminator cannot distinguish between synthesized examples and training examples any better than guessing [2, 3]. At this equilibrium the generator—the GAN—is ready for use.

GANs have been successfully applied in many fields; for example, drug design [4], galactic astronomy [5], and health care [6]. GANs have been applied most prominently and extensively to image processing, for image generation and image-to-image translation [2, 7, 8], to generate fake celebrity faces [9], for super-resolution [10], to give images the style of another image [11], and to selectively alter images [12]. These applications all involve inputs and outputs that are both high-dimensional features.^1^1In image processing the dimensionality of the GAN input is of the order of the image size in pixels. Generative networks are distinctive in this respect; the outputs of ANNs designed for classification or prediction are commonly just one-dimensional features, a categorical variable for ANN classifiers and a real-valued variable for ANN predictors. The high-dimensional problem settings in which GANs are applied complicate study of GAN performance because of the multitude of effects associated with the rich detail in the input during training and the challenge of assessing GAN performance from its high-dimensional distributional output. Additionally, the high dimensionality of both input and output imposes a computational burden on experiments with GANs. Directly addressing GAN performance in high-dimensional application settings has yielded only limited progress on pressing issues such as mode collapse [13], model evaluation [14], and training instability due to saddlepoints [15, 16], hidden low-dimensional target support [17], and absence of an equilibrium [18].

To avoid the complications entailed by high-dimensional inputs and outputs, we took a different tack and studied GANs in low-dimensional settings. This enabled us to transparently address some basic questions about GAN performance. In the work described in this paper, we conducted an experiment to explore the effects of training sample size and target distribution complexity on GAN error. Target distribution complexity is determined in significant part by the distribution’s number of modes. For example, consider the 28×28-pixel images of the 10 digits in the Modified National Institute of Standards and Technology (MNIST) data set [19]. The MNIST target distribution has nominally 10 modes within its 28×28-dimensional support, and its complexity can be varied by excluding digits from consideration. Modes—their placements, sizes, and feature associations—are not readily interpreted or manipulated in many high-dimensional applications, where mode shape^2^2The shape of a mode can be intuited in terms of the shape and dimensionality of the submanifold on which it resides; a mode in one dimension is a zero-dimensional point, and a mode in two dimensions might be a point or a section of a curve (on a one-dimensional sub-manifold). is a complicating factor. In low-dimensional settings, by contrast, multimodal target distributions are easily rendered, for example, by mixtures of Gaussian distributions.

This paper reveals important features of GANs and sets a path for further study. We focused on GAN performance in low-dimensional settings, where experiments can be conducted with a manageable computational burden. This approach also allows the experimenter to control for confounding effects and achieve transparent results. The experiment conducted in this study introduces and verifies procedures (for GAN error measurement, design, stability, and training convergence) that can be used in subsequent experiments with broader scope and more factors. Also, this study emphasizes the primacy of the relationship of GAN error to the size *N* of the training sample, and our experiment finds that GAN error is log-log linear with respect to *N*. Surprisingly, in the cases in our experiment, this relationship extends to even small *N*, allowing GAN error to be understood succinctly in terms of its error exponent (log-log slope). Finally, our experiment uncovers two forms of GAN error, which we term tail underfilling and bridge bias. This demonstrates that GAN mode tunneling (of which bridge bias is the low-dimensional analogue) occurs, and can be studied, in low-dimensional GANs. This is a validation of our choice to study GAN performance in low-dimensional settings.

The remainder of this paper is organized as follows. Section 2 presents important details of our experiment, including the experiment protocol followed and our use of earth mover (EM) distance to quantify GAN error *d*(*L, T*). We also present the architecture of the GAN in our experiment and our GAN training procedure. Section 3 presents and discusses the results of our experiment. Section 4 closes with a summary and some related final remarks.

## 2 Preliminaries

This section details the way our experiment was conducted. This includes, in particular, the protocol used for the experiment’s trials and the particular form of GAN studied. We also discuss our use of EM distance to quantify GAN error.

## 2.1 Protocol for Experiment Trials

The primary objective of our study was to determine how training sample size affects GAN performance for different degrees of target distribution complexity. Our experiment to explore this relationship among GAN error, sample size, and target complexity consisted of trials in which our GAN was trained and exercised over a range of training sample sizes *N* for each of six target distributions *T* with varying complexity, including three one-dimensional distributions and three two-dimensional distributions.

**Fig. 1:**
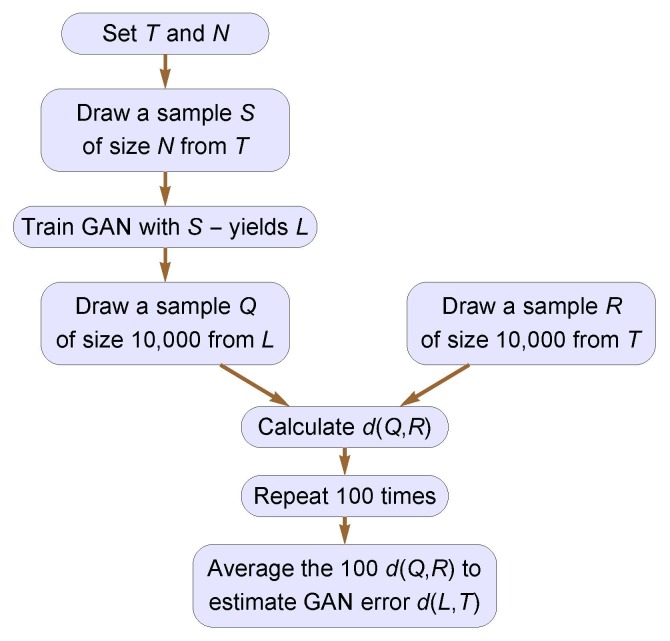
Protocol for trials in GAN performance experiment. The GAN learns *L* from a training sample *S*. The GAN error *d*(*L, T*) is then estimated by comparing samples *Q* and *R* from *L* and *T*, respectively.

All trials in our experiment were conducted according to the protocol diagrammed in Fig. 1. Each trial began with a given target distribution *T* and a size *N* for the training sample *S*. The training sample *S* was drawn by simple random sampling from *T* and used to train the GAN. Once trained, the GAN generator synthesized a sample *Q* of size 10,000 from the GAN’s learned distribution *L*, a second sample *R* of size 10,000 was drawn from *T*, and the distance *d*(*Q, R*) was calculated. This distance *d*(*Q, R*) is an approximation of the GAN error *d*(*L, T*). This whole process was repeated 100 times (a new GAN trained each time), and the GAN error *d*(*L, T*) for the trial was estimated by the average *d*(*Q, R*) of the 100 *d*(*Q, R*).

## 2.2 GAN Fundamentals

A GAN has two components in its training phase, its generator, *G_θ_*, and a discriminator, *D_ϕ_*, where *θ* and *ϕ* are vectors of parameters estimated in the learning process. The generator *G_θ_* is a mapping from a noise (latent) distribution *Z* to its learned distribution *L*. The discriminator *D_ϕ_* assigns a probability^3^3A discriminator that outputs a class membership probability rather than just a class label is sometimes termed a critic [20]. that a presented example, whether from *T* or synthesized, comes from *T* . Both *G_θ_* and *D_ϕ_* are typically implemented by ANNs. These two networks are trained according to a combined loss function [21]

*ℒ* (*θ,ϕ*) = *E_ζ_*_∼_*_Z_* [ *f* (*D_ϕ_* (*G_θ_* (*ζ*))] + *E_τ_*_∼_*_T_* [ *f* (−*D_ϕ_* (*τ*))]. (1)

At each iteration in training, *G_θ_* tries to minimize *ℒ* (*θ,ϕ*) while *D_ϕ_* tries to maximize *ℒ* (*θ,ϕ*). Popular GAN variants are distinguished by different choices of *f* (*x*) in Eq. (1). For example, the original Goodfellow GAN [2] and the Wasserstein GAN [20] correspond, respectively, to *f* (*x*) = −ln(1 + *e*^−^*^x^*) and *f* (*x*) = *x*. When the GAN discriminator is optimal, the GAN generator minimizes the distance between the target distribution *T* and the distribution *L* learned by the generator; the Goodfellow GAN minimizes the Jensen-Shannon divergence, and the Wasserstein GAN minimizes the EM distance between *L* and *T* . We used in our experiment a Wasserstein GAN with a gradient penalty term, called a WGAN-GP [22], added to the loss in Eq. (1). Table 1 summarizes the architecture of our WGAN-GP; this architecture is recommended in Ref. [24]. The generator output dimension is either one or two depending on the dimension of the target distribution in our experiment. We used an Adam optimizer [27] for both the generator and discriminator with a learning rate of 1e–4, with *β*_1_ = 0.5 and *β*_2_ = 0.9. For each update of the generator, we performed five updates of the discriminator. We found these values to give relatively consistent, stable results during training. Choosing training parameters remains an art based on experience and trial-and-error.

**Fig. 2:**
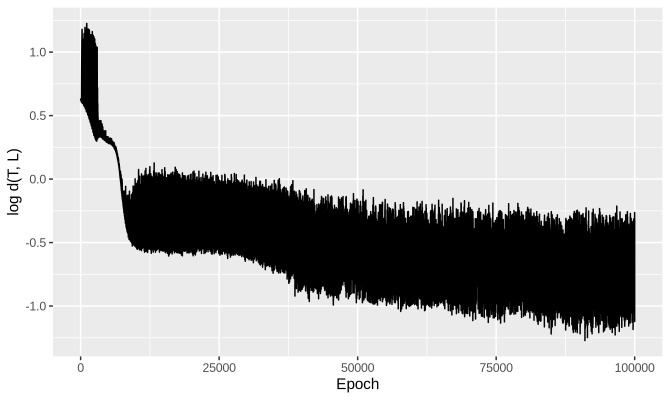
GAN error versus number of epochs for a representative experiment trial. In our experiment, the GAN was trained in each trial for 50,000 epochs.

The GAN training process is notoriously unstable, and various expediencies have been proposed in the literature to stabilize training [15]. These include changes to the loss function used in training [20, 22], tricks for improving GAN training [15, 23], and investigations of GAN convergence [24, 26]. Our WGAN-GP is known to exhibit training instabilities [24], but we encountered none in our low-dimensional setting. We found with the WGAN-GP that the GAN error *d*(*L, T*) consistently reached steady-state after training for 50,000 epochs.^4^4An epoch is one complete use of the training sample *S* during learning. Usually, only a subset (batch) of *S* is used in any given learning iteration, so one epoch typically corresponds to multiple iterations.
Figure 2 shows the evolution of GAN error with number of learning epochs for a typical trial in our experiment.

**Table 1:** Architecture of GAN generator (left) and discriminator (right). The generator input is a vector of independent N(0, 1) noises with a length of 128. The leaky-relu function is defined *f* (*x*) = 1(*x <* 0)(*αx*) + 1(*x* ≥ 0)(*x*), where *α* is a small constant.

	Generator			Discriminator	
Layer	Connectivity No. of units	Activation	Layer	Connectivity No. of units	Activation
1	Full 128	Leaky-relu	1	Full 32	Leaky-relu
2	Full 128	Leaky-relu	2	Full 32	Leaky-relu
3	Full 128	Leaky-relu	3	Full 32	Leaky-relu
4	Full Dim. of *T*	None	4	Full 1	Leaky-relu

## 2.3 EM Distance

Our study of GAN performance needed a measure of distance between distributions to quantify GAN error. EM distance is attractive for this purpose, and hereafter, *d*(*U,V*) denotes the EM distance between distributions *U* and *V* . EM distance is a special case (*p* = 1) of *p*-Wasserstein distance, which measures separation among probability distributions and is a metric in the general setting of Radon spaces [25]. Let *U* and *V* be two distributions on *d*-dimensional Euclidean space, where ℜ*^d^* is defned by cumulative distribution functions (CDFs) *F* and *G*, respectively. The EM distance separating the distributions *U* and *V* is

$d(U,V)=\underset{H}{\inf}\int_{{ℜ}^{d}x{ℜ}^{d}}||\vec{u}-\vec{v}||dH(\vec{u},\vec{v}),$;

where *H* is any joint CDF on ℜ*^d^* × ℜ*^d^* such that, marginally, *U* ∼ *F* and *V* ∼ *G*. EM distance can be understood intuitively as the infmum cost required to move/rearrange a probability mass distributed according to *U* ∼ *F* into the distribution of *V* ∼ *G*, with Euclidean distance ‖ · ‖ measuring the move required for each infinitesimal of probability mass. The joint CDF *H* in the infmum in Eq. (2) represents different possible plans for transporting each infinitesimal of probability mass from *U* to *V* .

EM distance has a long history, stretching back to Monge’s 1781 work in transportation theory [28]. Recently, EM distance has been used broadly in computer science, with applications to pattern recognition [29], image databasing [30], and content-based image retrieval [31]. Arjovsky *et al.* used EM distance to formulate the Wasserstein GAN [20] to address issues of mode collapse and vanishing gradients, two problems inherent in the original GAN framework [2] and of continuing concern. EM distance is just one of many distance measures that can be defined for probability distributions [32], and no consensus has yet emerged for measuring GAN error [33]. Among measures of distance between distributions, EM distance has a powerful and prevailing role in many fields because of its sensitivity to both amount of mass and to underlying metrical, or ground, distance. This feature of EM distance, its dual sensitivity to mass and distance, makes it attractive for our purposes as a direct measure of GAN error.

EM distance can be expressed analytically in some limited cases, and in one dimension the sample *S* closest in EM distance to *T* takes a simple form. In general, though, EM distance is found numerically using Sinkhorn’s algorithm to solve a regularized version of the basic optimal transport problem [34, 35]. We used the Python Optimal Transport Library [36] implementation of Sinkhorn’s algorithm in this work to calculate GAN error *d*(*L, T*).

## 3 Experiment Results

**Fig. 3:**
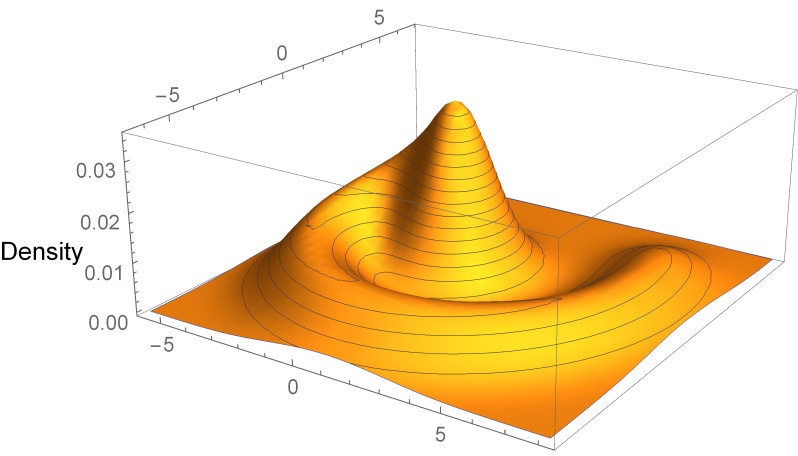
Density of the Swiss roll target distribution.

This section presents and discusses the results of our target distribution complexity experiment in which the relationship of GAN error to training sample size was studied as it varied with the complexity of the target distribution. The experiment included six target distributions, three one-dimensional distributions and three two-dimensional distributions. The three one-dimensional target distributions had one, two, and three modes. As remarked earlier, adding more modes can be thought of as adding more digits in the MNIST data set. The three two-dimensional distributions in this experiment were a Gaussian distribution, an equal mixture of two Gaussian distributions, and a Swiss roll distribution. The Swiss roll distribution, commonly used in machine learning studies [37], is given by the random vector

(*X*_1_, *X*_2_) = (*U* cos*U,U* sin*U*) + (*W*_1_*,W*_2_),

where *W*_1_, *W*_2_, and *U* are independent random variates with *U* ∼ Unif(0, 9*π/*4) and *W*_1_*,W*_2_ ∼ N(0, 0.01). The density of the Swiss roll distribution is shown in Fig. 3.

### 3.1 Results

**Fig. 4:**
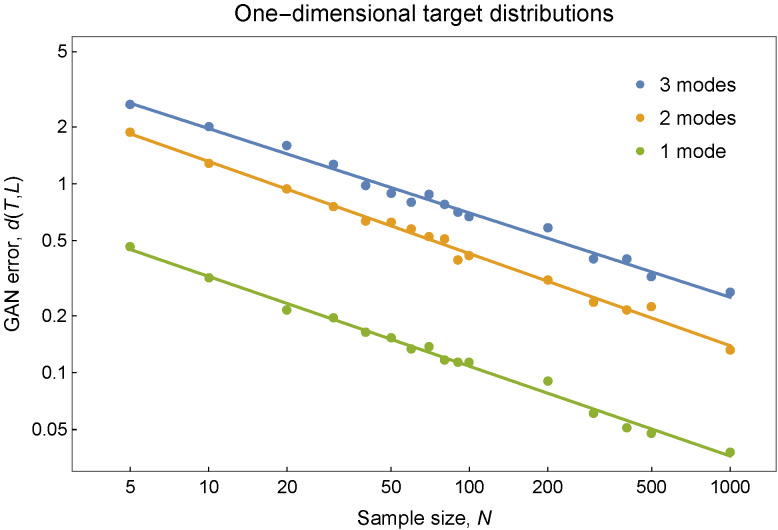

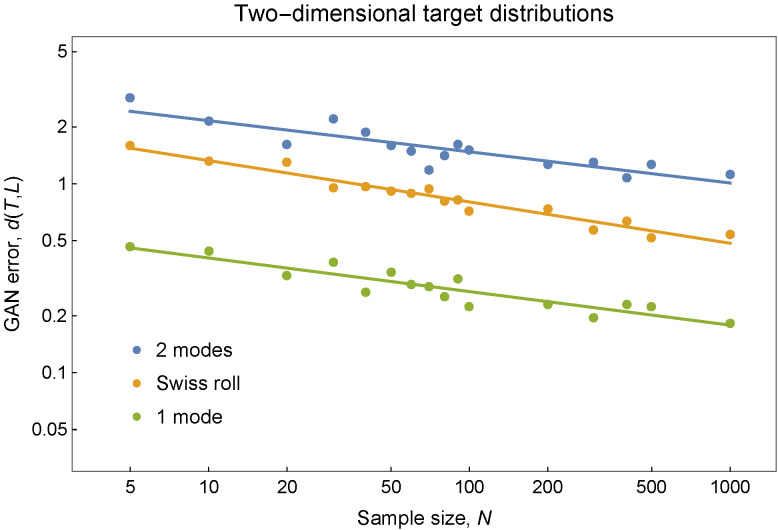
GAN error *d*(*L, T*) for one-dimensional target distributions with one, two, and three modes (top) and for three two-dimensional target distributions (bottom). For the one-dimensional *T*, the GAN error decreases with sample size *N* at a common rate with estimated error exponent 0.47. For two-dimensional *T*, the error decreases at a common rate with estimated error exponent 0.19.

The results from the target distribution complexity experiment are plotted in Fig. 4. First, these results confirm that increased target complexity results in poorer GAN performance and greater error. We see this in Fig. 4 for the one-dimensional *T* in the ordering of higher GAN error with greater number of modes. Figure 4 shows this also for the two-dimensional *T* in the experiment, provided the Swiss roll *T* is interpreted as more complex than a distribution with a single point mode but less complex than a *T* with two point modes. Second, in the log-log format of Fig. 4, the relationship of GAN error *d*(*L, T*) to sample size *N* appears to be linear

log *d*(*L, T*) = *a* − *b* log *N* (3)

or, equivalently, *d*(*L, T*) = 10*^a^N*^−^*^b^*, with error exponent *b*. This linear relationship might be anticipated to hold asymptotically for large *N*; in the results in Fig. 4, it appears to apply even at small sample sizes. Analysis of a standard analysis of covariance (ANCOVA) model confirms this. That analysis indicates further that the error exponent *b* in Eq. (3) differs significantly according to the dimensionality of *T*, but that no statistically discernible differences exist (at *α* = 0.05, general linear *F* test [38]) among the error exponents for the one-dimensional *T* or among those for the two-dimensional *T* distributions. The common error exponents are estimated to be 0.47 and 0.19, respectively, for the one- and two-dimensional *T* distributions in the experiment.

The GAN errors *d*(*L, T*) shown in Fig. 4 were obtained according to the protocol in Fig. 1. The protocol’s size, using 10,000 samplings of *Q* and *R* and its numerical approximation of *d*(*Q, R*) together impose a noise floor on the GAN error that can be resolved by this approach. This floor can be determined by using the protocol to find *d*(*L, T*) in the extreme case where the GAN learns the target distribution perfectly so that *L* = *T* with zero GAN error. These protocol-estimated *d*(*T, T*) floors are reported in Table 2 for each target distribution in our experiment. GAN errors below these floors cannot be accurately determined by our protocol with size-10,000 *Q* and *R* samples; larger sample sizes would lower these floors and increase the protocol’s resolution. The floors in Table 2 show that *Q* and *R* samples of size 10,000 are sufficient for our experiment.

The measurements of GAN error *d*(*L, T*) in Fig. 4 made according to the protocol in Fig. 1 were produced on the Raritan computing cluster at the National Institute of Standards and Technology. Twelve of the cluster’s Nvidia Volta graphics processing units (GPUs) were used in parallel for the experiment^5^5Certain commercial equipment, instruments, or materials are identified in this paper in order to specify the experimental procedure adequately. Such identification does not imply recommendation or endorsement by the National Institute of Standards and Technology, nor does it imply that the materials or equipment identified are necessarily the best available for the purpose.. Training a GAN to 50,000 epochs (Fig. 2) took about 45 min. By training 12 GANs in parallel, each GAN error *d*(*L, T*) in the experiment was calculated in 5 to 8 h. The six error curves in Fig. 4, each estimated from 16 error measurements, therefore required a total of about 600 h (25 d) to complete.

**Table 2:** Measurement floors for GAN error for the six target distributions in Fig. 4. These floors were estimated by *d*(*T, T*) with size 10,000 samples from *T* .

	GAN error
Target distribution *T*	measurement floor
One-dimensional distributions:	
Unimodal Gaussian	0.018
Bimodal Gaussian mixture	0.072
Trimodal Gaussian mixture	0.117
Two-dimensional distributions:	
Unimodal Gaussian	0.057
Swiss roll	0.080
Bimodal Gaussian mixture	0.042

### 3.2 Discussion

**Fig. 5:**
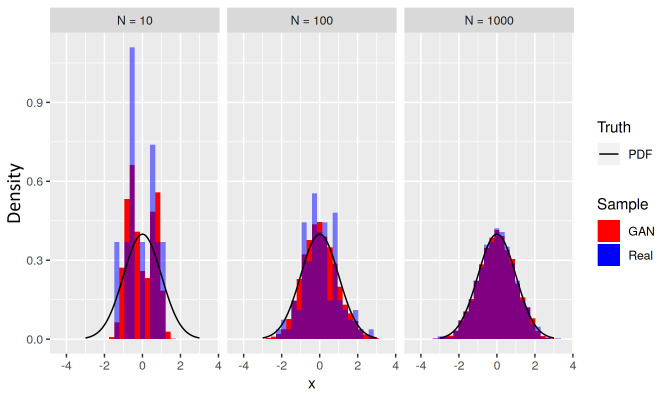
Histograms (red) of learned distributions *L* for a bimodal target distribution *T* (black), for three sizes *N* = 10, 100, and 1000 of training sample (blue). Each histogram of examples from *L* exhibits a bridge bias where the GAN has generated examples inconsistent with both the density of the bridge in *T* and the training data drawn from distribution *T* .

The low-dimensional setting of our experiment readily revealed two major sources for the GAN errors presented in Fig. 4: underfilled tails and bridge bias. Figure 5 presents three trials with a target standard normal N(0, 1) distribution, using training sample sizes *N* ranging from 10 to 1000. Fig. 5 shows that at small training sample sizes *N*, the GAN’s learned distribution, represented by the examples in red, underfills the tails of the target distribution (in black), and this error diminishes as *N* increases. Figure 5 also suggests that this is not inherently a problem with the GAN. The GAN can do no better than the training data available to it, and the poor fidelity of the training sample (in blue) at small *N* is at least partially the origin of the underfilled tails.

**Fig. 6:**
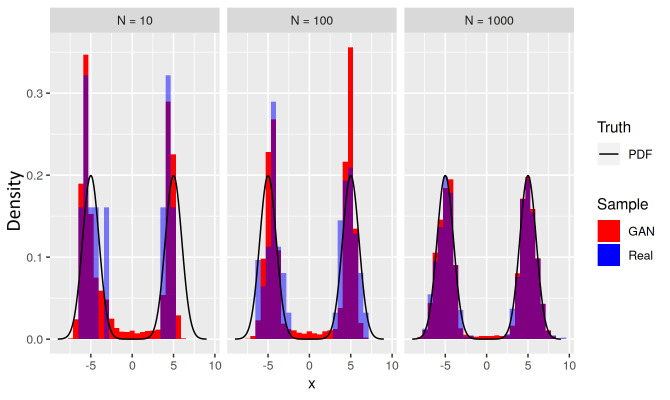
Histograms (red) of learned examples from distribution *L* for a unimodal target distribution *T* (black) for three sizes *N* = 10, 100, and 1000 of training sample (blue). The learned distribution *L* underfills the tails of *T* at small sample sizes *N*.

Figure 6 shows results of a GAN trained on a bimodal target distribution (in black) made of an equal mixture of two normal distributions N(−5, 1) and N(5, 1). The target distribution *T* has a low-density bridge connecting its two modes. Figure 6 shows that for small *N* the GAN’s learned distribution (examples in red) over-estimates the bridge density, even though the training sample (in blue) actually underrepresents the target bridge. The side experiment described next suggests that this bridge bias is a GAN structural feature that is only slowly corrected by increasing the size *N* of the training sample.

**Fig. 7:**
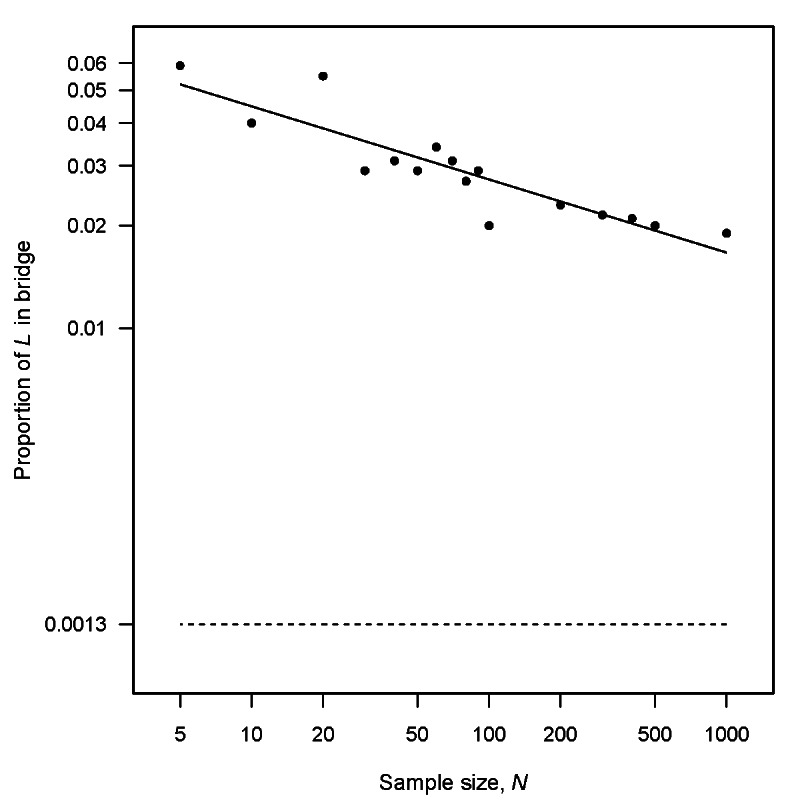
Bridge bias in the GAN-learned distribution *L* of the univariate bimodal target distribution *T* . The plotted proportions of *L* in the bridge of *T* decrease slowly with increasing training sample size *N*. The learned distribution *L* over-estimates the proportion of *T* in its bridge (dashed line).

A side experiment separate from our main target complexity experiment was conducted to explore the bridge bias seen in Fig. 6. This side experiment looked at only the univariate bimodal target distribution *T* of Fig. 6, estimating the size of the bridge in the GAN-learned distribution *L* over a set of training sample sizes *N*. The results of this side experiment are presented in Fig. 7, where the proportion of *L* in *T* ’s bridge^6^6The target distribution *T* is a bimodal mixture of Gaussian distributions N(−5, 1) and N(5, 1). We defined *T* ’s bridge to be that part of *T* that falls more than three standard deviations below its upper mode and more than three standard deviations above its lower mode. With this definition the bridge constitutes 0.0013 of *T* .
is plotted against *N*. These results show diminishing bridge bias as the training sample size increases. However, extrapolation of the regression line (solid line) in Fig. 7 suggests that very large sample sizes would be needed—even in this simple one-dimensional learning problem—to approach the target bridge proportion (dashed line) and effectively eliminate the bridge bias in *L*.

**Fig. 8:**
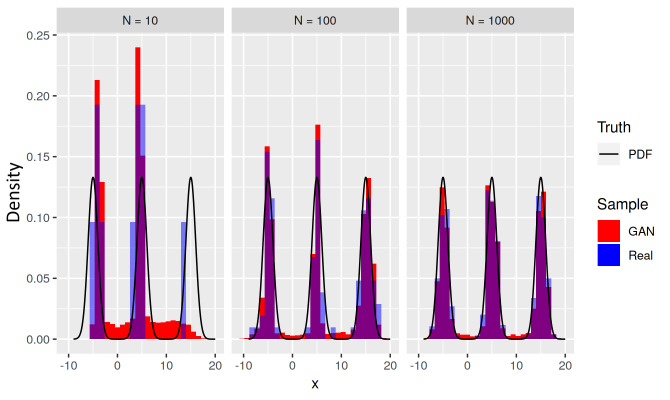
Histograms (red) of learned distributions *L* for a trimodal target distribution *T* (black), for three sizes *N* = 10, 100, and 1000 of training sample (blue). Each histogram of examples from *L* exhibits a bridge bias where the GAN has generated examples inconsistent with both the density of the bridge in *T* and the training data drawn from distribution *T* .

The trimodal target distribution in Fig. 8 shows that for a given sample size *N*, bridge bias worsens in the presence of multiple target bridges. Figures 6 and 8 show the presence of GAN bridge bias in examples where the target distribution modes are connected by bridges. In fact, GAN bridge bias occurs even when no bridge connects different modes of the target distribution, as demonstrated when the training sample happens to have no examples between two modes.

**Fig. 9:**
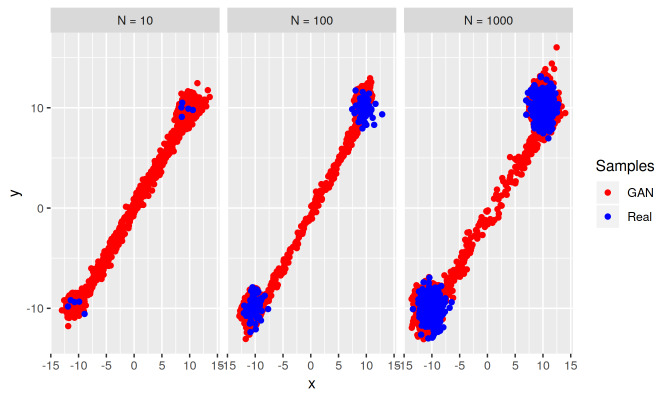
Scatterplots of GAN-generated samples (red) for the bimodal bivariate target distribution, for training samples (blue) of three sizes *N* = 10, 100, and 1000. The GAN exhibits a strong learned bridge bias.

The GAN bridge bias seen with our one-dimensional multimodal target distributions appears also with our two-dimensional distribution *T* . Figure 9 shows GAN-generated samples for the target bimodal mixture of bivariate Gaussian distributions in our experiment. In each of the three cases in Fig. 9—*N* = 10, 100, and 1000—the learned bridge between the two modes greatly over-estimates the (very low) density of the bridge in the target distribution. This bias is greater than that for our one-dimensional target distributions. Also, while the bridge bias for two-dimensional *T* decreases with *N*, this decrease is slower than that in the corresponding one-dimensional *T* . Interestingly, this bridge bias in two dimensions is restricted mostly to the one-dimensional line between the two modes, meaning that while the bridge bias for two-dimensional *T* is more pronounced compared to that for one-dimensional *T*, it is also more restricted relative to the support of *T* . Experiments to explore the relationships among amount and extent of GAN bridge bias, GAN architecture, training parameters, training sample size, and target complexity (dimensionality and multimodality) are needed.

**Fig. 10:**
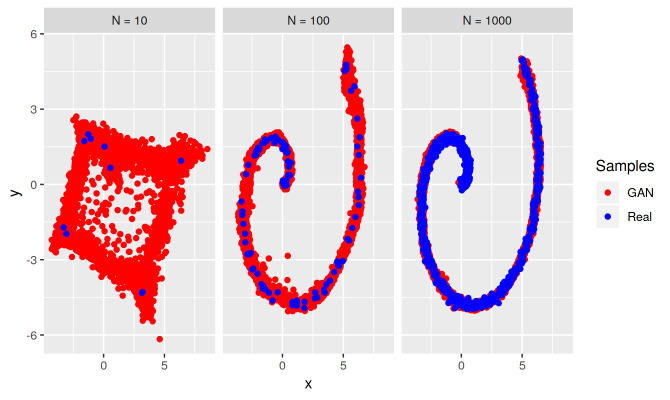
Scatterplots of GAN-generated samples (red) for the Swiss roll target distribution, for training samples (blue) of three sizes *N* = 10, 100, and 1000. The GAN exhibits a bridge bias for *N* = 10. Unlike for our other one- and two-dimensional target distributions, though, the GAN soon learns the essential structure of the Swiss roll as *N* is increased, and bridge bias no longer occurs.

The univariate and bivariate bimodal target distributions in Figs. 6 and 9 have point modes and the GAN bridge between the modes is essentially one-dimensional. The Swiss roll distribution in our complexity experiment affords us an opportunity to see what can happen when a target mode extends beyond just a point. Figure 10 presents scatterplots of GAN-generated samples for the Swiss roll target distribution in our experiment. The training sample of size *N* = 10 is not enough for the GAN to learn the submanifold on which the target ridge resides and it reverts to identifying modes with heavily biased bridges. With a relatively small training sample size *N* = 100, though, the GAN has discovered this structure and almost all bridge bias is gone. Bridge bias could be understood to arise from the fact that the generator is a continuous map from the latent space of the GAN input noise distribution to the support (data space) of the target distribution, while a bimodal target distribution with no bridge (zero mass between modes) would require a discontinuous map between the latent space and the data space. In other words, GAN bridge bias may be an artifact of a continuous approximation to a discontinuous function.

**Fig. 11:**
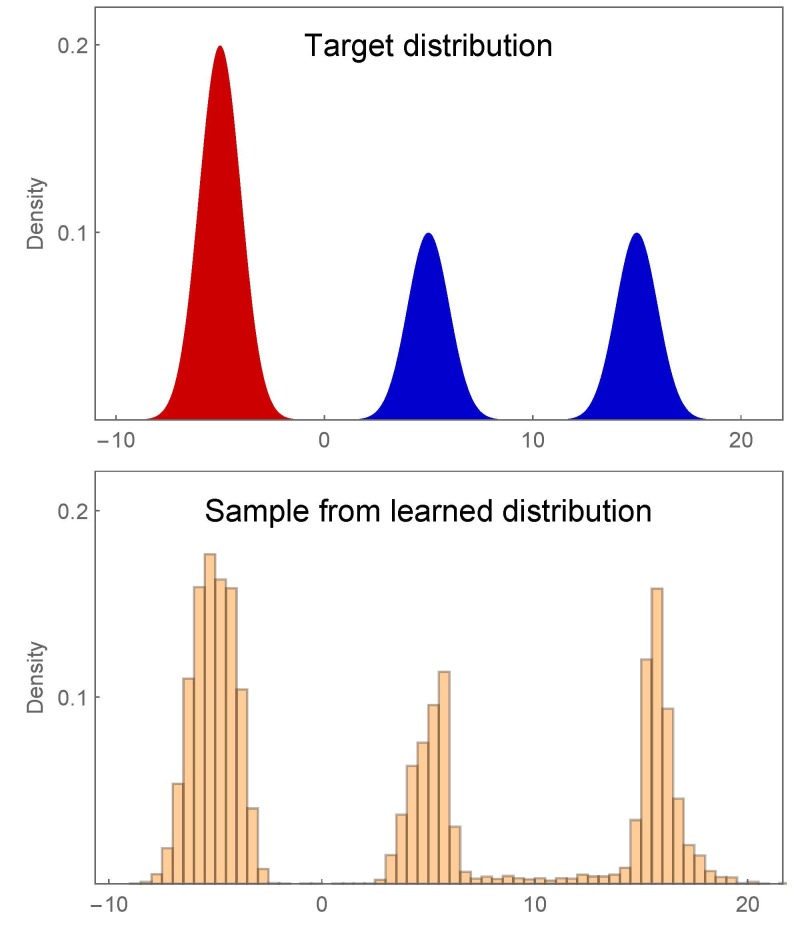
A trimodal target distribution (top) with color-labeled modes and data (bottom) synthesized by a conditional GAN. A bridge exists in the learned distribution between the center and right modes of the same labeled class but not between the left and center modes from different labeled classes.

Our experiment found that GAN performance decreases as modes are added, and we identified bridge bias as a significant cause. In fact, a GAN bridge can exist between modes even in the absence of any corresponding target bridge. These GAN bridges may be the source of low-fidelity realizations synthesized by GANs trained for image generation [39]. A conditional GAN can alleviate bridge bias to some degree. A GAN is trained in an unsupervised fashion, with no labels or other identifying information attached to the examples in the training sample *S*. A conditional GAN is trained with labeled data, and if these class labels line up well with target modes, bridge bias can be reduced. To see this, consider the trimodal target distribution *T* shown (top) in Fig. 11. Training examples drawn from *T* are identified (red or blue) depending on the mode from which they derive. The color labels unambiguously identify the left mode, but they do not resolve the center and right target modes. This is akin to having labels for images of cats and dogs, but no labels specifying dog breed. A GAN trained with labeled data—a conditional GAN [40]—learned the distribution shown (bottom) in Fig. 11. The left and center modes from different classes have no appreciable bridge, while the center and right modes from the same class have a bridge. Of course, labeled data are not always available, and when labels are available they may not correspond to different modes. Still, further study is warranted to discover when and to what extent a conditional GAN with labeled training data can alleviate bridge bias.

## 4 Summary and Related Remarks

GAN performance studies in the setting of high-dimensional applications have made only limited progress on pressing problems associated with GAN training, including mode collapse and training instability. In this study, to make progress on these and other questions relating to GAN performance, we took a fresh approach and considered GAN performance in low-dimensional settings. This approach offered important advantages: a reduced computational burden in experiments, more comprehensible and malleable target distributions, and easier assessment of GAN error. Our low-dimensional approach also carried risk. A low-dimensional GAN may not reproduce the high-dimensional phenomena that need to be understood. Encouragingly in this regard, our experiment revealed bridge bias in trained GANs, analogous to that seen in high dimensions.

A major purpose of our study was to establish protocols for GAN design and experimentation that fully exploit the advantages of low dimension and that can be used in subsequent, more elaborate experiments with low-dimensional GANs. Beyond this, our work makes two contributions. First, our work highlights the perspective of GAN error as a function of training sample size, because our experiment shows that this relationship is log-log linear and that the GAN error exponent (log-log slope) depends solely on the dimension of the target distribution. Second, our experiment uncovers two prominent forms of GAN error, tail underfilling and bridge bias, finding that both decrease only slowly with increasing sample size.

This initial study of low-dimensional GAN performance offers a framework for further investigation in many directions. Target distribution complexity can be varied by dimension and by number, distribution, and dimensionality of modes. Our experiment found, for example, that doubling the dimension of the target support from 1 to 2 roughly halved (from 0.47 to 0.19) the GAN error exponent. Determining whether and how this effect scales to higher dimensions has important implications for defining how well GANs can reasonably be expected to perform in many application settings. Also, investigation into the relationship between bridge bias and bridge length (spacing between modes) is needed; all else remaining equal, modes tend to be more separated in higher-dimensional data spaces.

All the GAN training samples in our experiment were drawn from the target distribution by simple random sampling. Other sampling schemes can be envisioned; one such scheme is stratified random sampling, in which the training sample is assembled from simple random samples drawn from each target mode. This higher-fidelity sampling scheme could be expected to yield reduced GAN error for any given sample size *N*; one would like to determine whether such a sampling scheme would, more powerfully, increase the GAN error exponent. The effect of training sample fidelity could be tested to its limit by studying training samples *S* that minimize the EM distance *d*(*S, T*) separating *S* and the target distribution *T* .
